# Supplementary material for: Signature of a continuous quantum phase transition in non-equilibrium energy absorption: Footprints of criticality on higher excited states
Source: Sci Rep. 2015 Nov 16;5:16490. doi: 10.1038/srep16490 (PMC4644967; doi:10.1038/srep16490)
Supplement: Supplementary Information [file srep16490-s1.pdf]

Supplementary Material for
Signature of a continuous quantum phase transition in non-equilibrium energy absorption: Footprints of criticality on higher excited states

Sirshendu Bhattacharyya
R.R.R. Mahavidyalaya, Radhanagar, Hooghly, India

Subinay Dasgupta
Department of Physics, University of Calcutta, 92 Acharya Prafulla Chandra Road, Kolkata 700009, India

Arnab Das
*Theoretical Physics Department, Indian Association for the Cultivation of Science, Kolkata 700032, India**

NONANALYTIC SIGNATURE OF CRITICAL POINT IN THE TRANSVERSE SPIN POLARIZATION

We shall now discuss the special case of $0 \to h_F \to 0$ pulse in the XY chain keeping anisotropy constant at γ. In this case, for $t < 0$ the z-component of spin polarization $M_z(t) \equiv \frac{1}{N}\sum_j s_j^z$ is zero due to symmetry and energy $E(t<0) = \langle\psi(t)|\mathcal{H}(t)|\psi(t)\rangle$. Thus,

$$E(t<0) = -\frac{N}{2}(1+\gamma)C_1^x - \frac{N}{2}(1-\gamma)C_1^y,$$

where $C_1^x \equiv < s_j^x s_{j+1}^x >$, $C_1^y \equiv < s_j^y s_{j+1}^y >$. Just after the quench the energy can be defined as $E(t=0^+) = \langle\psi(t=0^-)|\mathcal{H}(t=0^+)|\psi(t=0^-)\rangle$. Hence

$$\begin{aligned} E(t=0^+) &= N\left[-\frac{1}{2}(1+\gamma)C_1^x - \frac{1}{2}(1-\gamma)C_1^y - h_F M_z\right]_{t=0^-} \\ &= E(t=0^-) \end{aligned}$$

Hence, the energy absorbed at the first quench is zero. During $0 < t < \tau$, $M_z(t)$ increases and the contribution of correlations decreases, keeping the total energy constant. For large τ, $M_z(t)$ attains some steady value $M_z(\tau)$. After the second quench, the energy is

$$\begin{aligned} E(t=\tau^+) &= \left[-\frac{N}{2}(1+\gamma)C_1^x - \frac{N}{2}(1-\gamma)C_1^y\right]_{t=\tau^-} \\ &= E(t=\tau^-) + h_F N M_z(\tau) \end{aligned}$$

Thus, the energy absorbed at the second quench, which is also the total energy absorbed due to application of the pulse, is simply

$$E_{abs} = h_F N M_z(\tau)$$

in this case.

DECAY OF $E_{\rm abs}$ WITH PULSE DURATION

In the special case of the transverse Ising chain the absorbed energy approaches the steady state value as $\tau^{-3/2}$

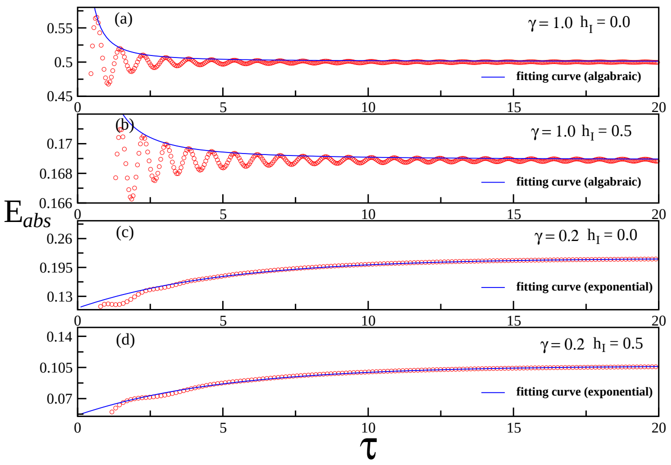

FIG. 1: **Decay of absorbed energy with pulse duration:** A field-pulse of critical value ($h_F = 1$) is applied keeping anisotropy constant ($\gamma_I = \gamma_F = \gamma$) : (a) For the transverse Ising model ($\gamma = 1$) starting from $h_I = 0$ & (b) for $h_I = 0.5$. In these cases the decay of the envelope is algebraic ($\sim \tau^{-3/2}$). (c) For the XY chain with constant anisotropy, ($\gamma = 0.2$) starting from $h_I = 0$ & (d) for $h_I = 0.5$. Here the decay is exponential irrespective of the value of initial field (h_I).

when a pulse having $h_F = 1$ is applied starting from any value of h_I. However in the case of the XY chain under the transverse field pulse of similar type, the absorbed energy decays exponentially with τ [Fig. (1)]. No such special behaviour is observed when a pulse of anisotropy is applied in the XY chain. [Fig. (2)]

ROLE OF HIGHER EXCITED STATES IN THE SIGNATURE:

Let $\{|\varepsilon_n^I\rangle\}$ and $\{|\varepsilon_n^F\rangle\}$ the complete set of orthonormalized eigenstates of $H(\lambda_I)$ and $H(\lambda_F)$ respec-

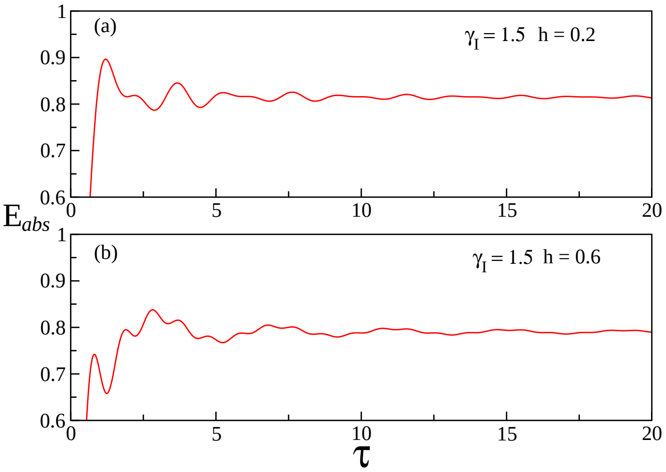

FIG. 2: **Decay of Absorbed energy with pulse duration :** A critical anisotropy pulse ($\gamma_F = 0$) is applied keeping the transverse field constant ($h_I = h_F = h$). Two cases are for two different h-values. Initial anisotropy (γ_I) is 1.5 in all the cases.

tively. Let also denote $\chi_{mn} = \langle \varepsilon^F_m | \varepsilon^I_n \rangle$. Taking the initial state to be the ground state of the Hamiltonian $H(\lambda_I)$, $|\psi(0)\rangle = |\varepsilon^I_0\rangle = \sum_n^{2^N} \chi_{n0} |\varepsilon^F_n\rangle$, we switch λ to λ_F at $t = 0$ and allow the system to evolve till $t = \tau$. The final wave-function is $|\psi(\tau)\rangle = \sum_n \chi_{n0} e^{-i\varepsilon^F_n \tau} |\varepsilon^F_n\rangle$. At $t = \tau$ as λ is switched back to λ_I, and the energy of the final state reads $\langle \psi(\tau)|H(\lambda_I)|\psi(\tau)\rangle = \sum_{n,k,m} \varepsilon^I_k \; [\chi_{n0} \; \chi^*_{m0} \; \chi_{mk} \; \chi^*_{nk}] e^{-i(\varepsilon^F_n - \varepsilon^F_m)\tau}$. For large N the spectrum is dense (almost continuous), and hence for large enough τ, all τ-dependent terms can be dropped out (Riemann-Lebesgue Lemma). This gives,

$$\langle \psi(\tau)|H_I|\psi(\tau)\rangle = \sum_k \varepsilon^I_k (\sum_m |\chi_{m0}|^2 |\chi_{mk}|^2). \qquad \text{(S1)}$$

The overlap $|\chi_{00}| = |\langle \varepsilon^F_0 | \varepsilon^I_0 \rangle|$ vanishes exponentially with N, and ε^I_0 grows at most linearly in N. Hence the "ground-state-only" quantity $|\chi_{00}|^4 \; \varepsilon^I_0$ vanishes as $N \to \infty$. Hence all the terms contributing to the non-analyticity at $\lambda_F = \lambda_C$ involve higher excited states.

Interpretation in terms of Ensembles produced by Projective Measurements:

Being the result of the complete dephasing approximation (no τ-dependent phase-term is kept), Eq. S1 has a simple physical interpretation. The final energy $\langle \psi(\tau)|H_I|\psi(\tau)\rangle$, as can be gleaned from Eq. (S1), is identical with the average of H_I over a mixed ensemble prepared in the following two-step measurement process (though the actual evolution is unitary). In the first step we prepare an ensemble of systems at the initial state $|\varepsilon^I_0\rangle$ and then measure H_F on the ensemble (i.e., do projective measurement in the $\{|\varepsilon^F_0\rangle\}$ basis. This produces a mixed ensemble $\rho_F = \sum_m |\chi_{m0}|^2 ||\varepsilon^F_m\rangle\langle|\varepsilon^F_m|$, where $|\chi_{m0}|^2$ is the probability of finding the system in the state $|\varepsilon^F_m\rangle$ when H_F is measured on the system at the state $|\varepsilon^I_0\rangle$. In the next step we carry on another projective measurement on ρ_F, this time on the eigen-basis of H_I. The ensemble produced after the second measurement is $\tilde{\rho} = \sum_k \left(\sum_m |\chi_{m0}|^2 |\chi_{mk}|^2\right) |\varepsilon^I_k\rangle\langle\varepsilon^I_k|$. Taking average of H_I over $\tilde{\rho}$ is clearly equal to $\langle \psi(\tau)|H_I|\psi(\tau)\rangle$ given in Eq. (S1).

ENERGY ABSORBED IN A SINGLE QUENCH

Here we show that for a single quench $H(h_I, \gamma_I) \to H(h_F, \gamma_F)$ (starting from the ground state of $H(h_I, \gamma_I)$), E_{abs} does not exhibit any non-analyticity for the following reason. For a single quench, it has the general form $E_{\text{abs}} = \dfrac{i}{2\pi} \mathcal{A}_1 \oint_C \dfrac{S(z, h_I, \gamma_I, h_F, \gamma_F)}{z\sqrt{Q^I(z, h_I, \gamma_I)}} \, dz$, where $\mathcal{A}_1 = \dfrac{1}{\sqrt{(1-\gamma_F^2)}}$; $S = \gamma_I(\gamma_I - \gamma_F)(z^2-1)^2 - 2(h_I - h_F)(z^3 + 2h_I z^2 + z)$ and $Q^I = (z - z^I_1)(z - z^I_2)(z - \frac{1}{z^I_1})(z - \frac{1}{z^I_2})$ with $z^I_{1,2} = \dfrac{1}{1-\gamma_I}[-h_I \pm \sqrt{h_I^2 + \gamma_I^2 - 1}]$. Clearly, in this case the only pole is at $z = 0$, which is independent of parameters of the Hamiltonian, and hence no non-analyticity is observed as a function of λ_F.

---

* Electronic address: arnab.das.physics@gmail.com
